# Searching for a Role of Nursing Personnel in Developing Landscape of Ehealth: Factors Determining Attitudes toward Key Patient Empowering Applications

**DOI:** 10.1371/journal.pone.0153173

**Published:** 2016-04-06

**Authors:** Mariusz Duplaga

**Affiliations:** Department of Health Promotion, Institute of Public Health, Faculty of Health Sciences, Jagiellonian University Medical College, Krakow, Poland; Institute for Health & the Environment, UNITED STATES

## Abstract

**Introduction:**

Nurses may play an important role in the delivery of medical services based on the use of ehealth tools. Nevertheless, their taking an active role in an ehealth environment depends on their possessing the appropriate skills and mindset. The main objective of this paper was to assess nurses’ opinions and to analyze the predictors of their acceptance of ehealth features relevant to patient empowerment with a strong focus on chronic care.

**Methods:**

A survey was conducted among nurses from hospital centers of south-eastern Poland based on a questionnaire designed to assess their attitudes toward the ehealth domain. The predictors of the nurses’ acceptance of ehealth usage within specific contexts were assessed with uni- and multivariate logistic regression.

**Results:**

An analysis was performed on data from 648 questionnaires retained after a quality check. The duration of Internet use was consistently related to higher acceptance of ehealth applications and more certainty regarding the reliability of health-related information available on the Internet. Nurses from urban medical centers were more skeptical about the use of specific ehealth solutions.

**Conclusion:**

Previous experience in using information technologies is the main factor influencing the acceptance of specific ehealth solutions relevant for care provided to patients suffering from chronic conditions.

## Introduction

Presently, the use of information and communication technologies (ICT) is one of the key elements transforming health care services. During the beginning of the 21st century the term “ehealth” was introduced in order to name “an emerging field resulting from the intersection of medical informatics, public health and business, referring to health services and information delivered or enhanced through the Internet and related technologies” [1]. Originally ehealth also referred to the development of commerce on the Internet [2]. Ehealth was differentiated from telemedicine as a broader and more open approach to the use of ICT for health-related purposes, less hardware-centric [3] and driven more by non-professionals, especially patients, than by medical professionals [4]. Usage and an understanding of ehealth evolved quickly. In a study from 2005, Pagliari et al. were able to identify as many as 36 different definitions of ehealth [5]. In a short time, “ehealth” became a universally accepted term covering many areas related to the use of ICT in relation to health and health care. This broad understanding of ehealth was encapsulated by a definition proposed in 2004 by Jai Ganesh who understood ehealth as being “any use of electronic information and communication technology to promote health or improve health care” [6]. In line with this approach, the definition available on the webpage of the World Health Organization simply states that ehealth means “the use of ICT for health” [7].

Although many authors have attempted to provide a framework for ehealth (usually for the purpose of evaluating its impact) [8], a broad classification of available ehealth solutions or applications remains unavailable. Judging by examples provided by some authors, the range of possible solutions spans from; health-related Internet websites designed for various groups of users, telemonitoring and telecare solutions for chronic patients and elderly people, applications supporting interactions between patients and health professionals as well as systems supporting the workflow of health care institutions, including electronic health records and communication between organizations active in health services [9–11].

In most countries chronic diseases are the source of a growing burden to health care systems. Enhancing the efficiency of care delivered to patients with long-term medical conditions remains at the top of ehealth interests. The development of ehealth-based services may be an effective response to many challenges resulting from the growing number of chronic patients living in modern societies [12–13]. The systematic review of systematic reviews from 2014 yielded promising results concerning the effectiveness and cost-effectiveness of ehealth interventions in somatic diseases [14].

The concept of empowerment in relation to health stems from the classical definition of health promotion formulated by the Ottawa Charter stating that it is “a process of enabling people to increase control over and to improve their health” [15]. Although in this definition the word “enabling” was used, the term “patient empowerment” was later popularized in health behavior literature [16]. According to Johnston Roberts “patients are empowered when they have the knowledge, skills, attitudes, and self-awareness necessary to influence their own behavior and that of others to improve the quality of their lives” [17]. In relation to patients, empowerment means recognition of the importance of the patient’s own self-reliance in tackling the burden of disease [18]. It also relates to a new understanding of the patient-physician relationship–one which should be built on a partnership rather than a patronizing communication coming solely from the physician. Patient empowerment results in ability to participate in this partnership and make decisions about health [19–20]. More recently, van Uden-Kraan et al. related patient empowerment to the belief in patient autonomy and defined it as “the right and responsibility of patients to access health information and to make their own health-related decisions” [21]. A comprehensive definition of patient empowerment was proposed by Small et al. [22]. These authors stated that it is “an enabling process or outcome arising from communication with the health care professional and a mutual sharing of resources over information relating to illness, which enhances the patient’s feelings of control, self-efficacy, coping abilities and ability to achieve change over their condition”.

Patient empowerment has become an important motivating factor behind policies developed in many countries. Furthermore, ehealth is also indicated as a key strategy for developing patient empowerment. Key indicators of patient empowerment involve in the area of patient capacities: self-efficacy, knowledge, skills and attitudes, perceived personal control over health and healthcare, health literacy and in the area of behavior: participation in shared decision-making, management of own health and/or care and finally, the use of new technology to collect health information as well as the ability to participate in support groups [23].

At least several of these indicators may be mapped to functions or applications available within the ehealth environment. Monteagudo & Moreno indicated several mechanisms to help implement patient empowerment with ehealth. They include communication with health agents and services, health information access, involvement in a health education process, decision making aids, self-care support and chronic care integrated services support [24].

According to Alpay et al. there are four empowerment areas relevant to ehealth: insight into one’s own health condition, making informed consent, engaging in self-care activities and developing self-care habits as well as having the possibility to live independently [25]. Ehealth applications and solutions which may support these areas include access to personal electronic health record, access to online information and online decision-making aids, self-monitoring of health status and disease symptoms with telemonitoring tools and tools enabling management of disease with support from formal and informal care providers and services [25].

Indeed, secondary evidence seems to indicate that ehealth solutions may improve patient empowerment. The results of a systematic review published by Samoocha & al. in 2010 demonstrated that web-based interventions had a significant positive effect on empowerment when measured with the instruments appropriate for specific medical condition [26]. In turn, a systematic review prepared by Kuijpers et al. demonstrated that web-based interventions may be an effective tool for increasing patient empowerment in cancer survivors [27]. According to Vance et al, the use of their personal health record encourages patients to take a more active role in their own care [28].

The perception of the ehealth domain in Poland corresponds to how it is perceived in other countries. Nevertheless, the development of an ehealth environment remains limited in Poland. Although there are many Polish websites providing health-related content for patients and the public, the use of ehealth tools enabling access to health services is not widespread. Furthermore, medical institutions are also slow to institute ehealth solutions. A report prepared by the Institute for Prospective Technological Studies revealed that the level of ehealth deployment in Polish hospitals is one of the lowest in Europe [29]. Moreover, the use of ehealth applications is rare among Polish health professionals. The results of the European study published in 2008 revealed that all indicators related to ICT use by general practitioners were below the average level in Europe [30], even though nearly all physicians in Poland were Internet users [31].

An emphasis on development of ehealth-based services in Poland increased after accession to the European Union. Access to structural funds available for new member states enabled the creation of more ambitious plans for building an ehealth infrastructure. Ehealth became one of the priority areas for projects funded from these sources available either on regional or national levels. Plans to develop a national ehealth infrastructure were prepared by the Centre of Health Information Systems, an institution affiliated with the Ministry of Health [32].

Nurses are the largest group of health care professionals, and successful implementation of ehealth solutions, especially those involving patients and private citizens as end-users, cannot be achieved without their active participation. In fact, nurses are one of the first groups to embrace ehealth solutions to provide care [33]. According to the Canadian Nurses Association (CNA) “nursing practice in telehealth should be part of an integrated health-care service and should enhance existing health-care services by improving their accessibility, appropriate use and efficiency” [34]. In turn, the representatives of the International Council of Nurses (ICN) remarked that the crucial position nurses hold in advancing ehealth solutions results from their key roles as information brokers and coordinators of health care [35]. It is worth mentioning that the implementation of ICT solutions in health care is also perceived as one of the measures which could help alleviate the impending nursing shortage [36]. Among guidelines developed within the ENS4Care project funded by the European Commission in the range of research and development framework program (CIP-ICT-PSP-2013-7) there is a recommendation that nurses involved in preventive and other health interventions should have “the knowledge, skills, opportunities and capacity to use the tools and technologies effectively” [37].

It is obvious that the cooperation of a multidisciplinary team which includes nurses is essential for successful management of chronic diseases [38–39]. Frequently, nurses are the first to provide feedback to patients in case of doubts and inquiries about their health and health-related issues. With a growing number of ehealth solutions with feasible uses for patients with chronic disease, there is also an increasing need to provide educational interventions related to ehealth for the patient. It is the nurses who are frequently expected to provide first line response to patients using ehealth solutions [40]. Such instructional interventions can be carried out by nurses interacting with patients, but only if the nurses themselves understand and accept the use of ehealth applications. On the other hand, due to their key role in providing health care services, nurses are obliged to use various types of computer applications at work anyway [41–42]. Growing expectations surrounding the involvement of nursing personnel in handling ehealth applications is a new challenge for many professionally active nurses [43–45].

Although current undergraduate nursing programs include training in computer skills, information technology (IT) competencies of professionally active nurses are not high in Poland. The role of Polish nurses in implementation of ehealth development plans formulated on a national level is not clearly described [32]. It seems as well that nurses’ perceptions about their own role in developing an ehealth environment are diversified among themselves [46]. There are no sources regarding the use of health applications by Polish nurses, but it is obvious that every day practice requires them to at least use software installed in their specific institutions or units (e.g. hospital information systems or systems designed for ambulatory care). Development of advanced ehealth applications should be aligned with acceptance of all potential users and in this context, the opinions and readiness to accept and apply new solutions among nursing personnel is of key importance. In this paper, the attitudes of professionally active nurses employed in hospital centers located in urban and rural areas toward ehealth applications increasing patient empowerment were assessed. The rationale for this study was based on an understanding of the potential benefits resulting from implementation of ehealth services in chronic care, as well as an appreciation of the role of nursing personnel in this process. The author assumed that the attitude of nurses toward development of an ehealth environment may be a key factor influencing the success of involving patients in the use of specific applications. As ehealth is perceived as a domain which can increase patient empowerment, the opinions of nurses about four relevant ehealth applications were assessed.

The analysis was based on the results of a survey prepared as an assessment tool for studying general usage of IT among nurses, as well as their perception and acceptance of ehealth solutions, especially in the context of chronic care. The survey was carried out in selected hospital centers located in the south-eastern part of Poland. Apart from making a general assessment of nurses’ opinions about ehealth, this paper was aimed at analyzing factors which could predict acceptance or lack of the same in relation to specific ehealth solutions generally perceived as having an impact on patient empowerment. Specific research questions were focused on the influence of age, location of the hospital in which the respondent was employed as well as the respondent’s number of different places of employment. Among the general population, the use of IT is higher among younger users coming from larger urban centers and most often among those which are more active and mobile. The analysis performed in this study was designed to assess if the same trends are seen among nurses in relation to specific types of ehealth applications.

## Methods

### Survey

The survey was based on the use of self-administered questionnaires developed with the aim to assess information technology skills and usage as well as to explore opinions about the ehealth domain among nursing personnel. The questionnaire consisted of items designed to assess the nurses’ usage of IT (use, motivations, duration and frequency of computer and Internet use) including the use of information systems within the workplace, opinions related to the use of IT in health care (its feasibility, importance for the accomplishment of nurse’s duties, possibility of use for support of chronic care and long term-patients), opinions about professional groups and stakeholders that play roles as initiators of ehealth development, about beneficiaries as well as about rationale and main barriers to its development. Furthermore, their opinions about the importance of ehealth solutions in chronic care were explored. In the questionnaire, the items seeking for respondent’s opinions provided response options following the 5-point Likert scale and ranged from “strongly disagree” to “strongly agree” with the neutral response in the middle position. The initial results of the survey performed with this questionnaire from a limited sample of nurses working at urban hospitals were published earlier [46].

The results reported in this paper come from a survey carried out among nurses employed in several hospital centers located in two voivodeships in south-eastern Poland during the period from November 2011 to January 2013. The involvement of a facility was dependent on the consent coming from the management of the individual centers. If permission was granted, the questionnaires were distributed by a contact person working in the hospital. The same individual collected the completed questionnaires and delivered them to the Department of Health Promotion, Institute of Public Health, Jagiellonian University Medical College in Krakow.

Appropriate representation of urban and rural areas was assured. Basically, hospital centers located in Krakow (population surpassing 800,000 inhabitants) were assumed to represent an urban area and centers situated in other locations in Podkarpackie and Malopolskie Voivodships (surrounded by typical rural areas) represented rural areas. Hospital centers considered as being rural were located in cities of Krosno, Nowy Targ, Tarnow, Iwonicz Zdroj and Rymanow Zdroj.

The study obtained approval from the Bioethical Committee of Jagiellonian University in Krakow, Poland (opinion No. KBET/226/B/2011 issued October 27, 2011). The questionnaires were filled anonymously. If the respondent returned a completed questionnaire, informed consent was implied.

The analysis presented in this paper focused on the respondents’ opinions on ehealth applications supporting patient empowerment which include: electronic communication between patient and physician, Internet-based access to electronic health record for patients and telemonitoring. Furthermore, the nurses’ perception of the reliability of health-related resources on the Internet was analyzed. Potential factors influencing these opinions were assessed with appropriate statistical models.

### Statistical analysis

Statistical analysis was conducted with SPSS v.21 for Windows (Armonk, NY, USA). The descriptive statistics were calculated for variables included in the analysis. The frequencies for categorical values were provided after exclusion of the missing values. The influence of factors characterizing the study population on the attitudes to key ehealth services was assessed by univariate and multivariate logistic regression models. Data set used in the analysis is available as S1 File.

The dependent variables were derived from four items of the survey examining respondents’ opinions about the reliability of Internet-based health resources, the feasibility of Internet/e-mail communication between a physician and a patient, patients having Internet-based access to their medical records and the use of telemonitoring for patients with chronic diseases.

These survey items were formulated as follows (translation of items included in the Polish version of the questionnaire for referential purposes only):

- “Information available on the Internet related to medical conditions and their therapies is usually reliable”
- “Physician may provide advice to a patient via Internet/e-mail”,
- “Patients should have Internet-based access to his/her medical record”
- “Would you recommend the use of an Internet-based system for monitoring of disease, e.g. bronchial asthma or congestive heart failure to a person from your close family? “

Initial responses to these items, collected according to the five-point Likert scale, were collapsed into two categories: ‘1’ (“agree”)–if the respondent selected “strongly agree” or “rather agree” and ‘0’ (“other opinion”)–if the respondent selected “strongly disagree”, “rather disagree” or “not sure/I don’t know”. The decision to convert initial variables taken with the Likert scale to binomial variables was made after considering that the main aim of the analysis was the assessment of the importance of potential predictors for agreeing (accepting) opinions toward specific types of ehealth applications in comparison to other varying opinions. The main focus was on contrasting responses which could be described as “acceptance” or “lack of acceptance” (the latter resulting from negative opinion or other reasons such as insufficient knowledge or lack of opinion). Such an approach could enable better understanding of circumstances that may favor a more acceptant attitude among nurses in relation to usage of specific ehealth applications. Collapsing categories resulting from the use of the Likert scale and then applying a logistic regression model may potentially lead to information loss, however, in this case eliciting differences between all five categories was not a priority. Furthermore, such an approach allowed avoidance of potential difficulties in interpretation of results of multinomial logit or ordinary regression models [47].

The independent variables included in the model were the respondent’s gender, age, duration of Internet use, number of employment sites (one or more), place of main employment (hospital or other), and the location (urban or rural) of the hospital center where the questionnaire was collected. The age of respondents was used as a categorized variable with four intervals obtained after applying quartiles for the initial continuous variable. The four categories established for age included the following intervals: ≤35, >35 to 42, >42 to 47, and >47. The variable designated as “duration of Internet use” applied in the regression models was derived from a variable indicating Internet use and a variable related to duration of Internet use. Five values were assumed: “0”–for respondents who did not use the Internet at all or had used it for a period not surpassing 1 year, “1”–for duration of use from >1 to 2 years, “2”–for duration of use from >2 to 5 years, “3”–for duration of use from >5 to 10 years, and “4”–for a duration longer than 10 years. The use of a combined variable addressing the use of the Internet and its duration was dictated by requirements resulting from the use of multivariate logistic regression modeling in relation to the number of cases and the number of predictors used in model.

Since the age of respondents and years of professional experience were closely interrelated, and there were more missing values in the latter variable, the respondents’ age was used in the modeling. Furthermore, due to the interrelation of computer use and Internet use, and close relation of outcome variables having an Internet context, only the latter variable was retained in the logistic regression models.

In the first stage, the influence of predictors on the outcome variables was assessed with univariate logistic regression models. Then, multivariate models were developed, including all six predictors used for the univariate models. The multivariate logistic regression was preceded by multicollinearity diagnostic analysis with a calculation of variance inflation factor (VIF) values for independent variables. No concerns were raised, since all VIF values were below 2.0. For all independent variables, odds ratio (OR) and 95% confidence intervals (95%CI) were provided.

## Results

### Characteristics of the study group

The questionnaires were distributed to 800 nurses employed in selected hospital centers. Completed questionnaires were returned by 716 of them. After a quality check, 68 of the returned questionnaires were excluded from further analysis. The respondents were 96.9% (n = 628) female. The average age of respondents (n = 624) was 40.8 (standard deviation [SD] = 9.49) and the average duration of professional experience (n = 592) was 19.1 (SD = 10.2) years. A total of 87.2% (n = 565) of the respondents were employed in only one workplace, and for 79.6%, their main place of employment was a hospital. The percentage of respondents employed in centers classified as located in a rural area was 54.8% (n = 355).

Among the respondents, 91.5% (n = 593) were computer users and 82.9% (n = 537) Internet users. The duration of computer use was less than 5 years for 35.5% (n = 207) of the computer users. A total of 55.9% of Internet users reported using it for more than 5 years. The frequencies for the independent variable used in logistic regression models are included in Table 1.

**Table 1:** Characteristics of the study group.

Variable	n	%
Sex		
Female	628	96.91
Male	20	3.09
Age (years)		
≤ 35	170	27.24
> 35 to 42	169	27.08
> 42 to 47	132	21.15
> 47	153	24.52
Number of employment sites		
1	565	87.19
>1	83	12.81
Main place of employment		
hospital	516	79.63
other than hospital	132	20.37
Location of medical center		
rural	293	45.22
urban	355	54.78
Duration of Internet use		
no use or ≤ 1 year	111	17.43
>1 to 2 years	45	7.06
>2 to 5 years	187	29.36
>5 to 10 years	201	31.55
> 10 years	93	14.60

The variables of use of computer and the Internet were assessed in relation to sex, age category, number of places of employment, main employment site (hospital or other) and location of the medical center (rural or urban). The use of computer was significantly higher among younger respondents (chi^2^ Pearson test, p<0.001) and those from urban medical centers (Fisher exact test, p = 0.002). The use of the Internet was more frequent among younger nurses (chi^2^ Pearson test, p<0.001), those employed in more than 1 place (Fisher exact test, p = 0.001) and in urban medical centers (Fisher exact test, p = 0.009). Detailed results of the analysis of the impact of respondents’ characteristics on IT use are presented in Table 2.

**Table 2:** Factors influencing computer and Internet usage.

Variable	Computer use		Internet use	
	% (n)	p*	% (n)	p*
Gender				
Female	91.4% (574)	0.99	82.8% (520)	0.99
Male	95.0% (19)		85.0% (17)	
Age (years)				
≤ 35	97.1% (165)	<0.001	90.6% (154)	<0.001
> 35 to 42	94.1% (159)		88.8% (150)	
> 42 to 47	90.2% (119)		80.3% (106)	
> 47	83.7% (128)		69.3% (106)	
Number of employment sites				
1	91.0% (514)	0.29	81.1% (458)	0.001
>1	95.2% (79)		95.2% (79)	
Main place of employment				
hospital	90.7% (568)	0.16	82.8% (427)	0.99
other	94.7% (125)		83.3% (110)	
Location of medical center				
rural	87.7% (257)	0.002	78.5% (230)	0.009
urban	94.6% (336)		86.5% (307)	

*Chi^2^ Pearson or exact Fisher test

### Opinions about ehealth services

More than half the respondents (54.6%) expressed the opinion that health-related resources available on the Internet are usually reliable. Only 13.4% of them disagreed with this opinion and 32.1% did not have a clear opinion. The percentage of respondents opposed to the use of the Internet/e-mail by a physician to provide advice to a patient reached 40%. Nearly the same percentage supported the use of such a mode of communication between a patient and a physician (38.0%). Patients being able to access their Internet-based medical record was accepted by 60.7% and rejected by only 25.2% of the respondents. Finally, only 27.7% of the respondents would recommend the use of an Internet-based monitoring system to a close family member, 20.4% had no opinion and 51.9% would not recommend such an approach. Initial distribution of the responses to items used for developing dependent variables is shown in Table 3.

**Table 3:** Distribution of responses to key items from the survey used for development of outcome variables in the logistic regression models.

Variables	strongly disagree % (n)	disagree % (n)	not sure/I don’t know % (n)	agree % (n)	strongly agree % (n)
Information available on the Internet related to medical conditions and their therapies is usually reliable (n = 636)	2.7 (17)	10.7 (68)	32.1 (204)	47.2 (300)	7.4 (47)
Physician may provide advice to a patient via Internet/e-mail (n = 639)	11.3 (72)	26.8 (171)	23.8 (152)	29.3 (187)	8.9 (57)
Patient should have Internet-based access to his/her medical record (n = 647)	5.3 (34)	19.9 (129)	14.1 (91)	32.5 (210)	28.3 (183)
Would you recommend the use of an Internet-based system for monitoring of disease to a person from your close family? (n = 647)	5.6 (36)	22.1 (143)	20.4 (132)	34.0 (220)	17.9 (116)

### Predictors of opinions about ehealth services

Univariate logistic regression models revealed that the opinions about reliability of health-related resources on the Internet depended on the age of the respondent, location of the medical center and duration of Internet use (Table 4). Interestingly, older respondents were more inclined (odds ratio [OR] and 95% confidence interval [95%CI] for differences between the youngest and older age groups of 2.40 1.55–3.72, 1.75 1.10–2.78, and 1.75 1.12–2.74, respectively) and those employed in the medical centers located in urban areas (OR = 0.71, 95%CI: 0.52–0.98) were less inclined to trust health-related content on the Internet. Positive assessment of the reliability of such health-related Internet content was higher among respondents who had used the Internet for longer periods (OR, 95%CI for consecutive response options, 2.49, 1.53–4.05, 2.14, 1.53–3.46, and 2.10, 1.19–3.70, respectively).

**Table 4:** The results of univariate and multivariate logistic regression modeling for opinions about the reliability of health-related resources on the Internet and about the use of Internet/e-mail for communication between a physician and a patient.

Variable	Information related to medical conditions and their therapies available on the Internet is usually reliable^#^ (‘agree’ vs. ‘other opinion’)				Physician may provide advice to a patient via Internet/e-mail^#^ (‘agree’ vs. ‘other opinion’)			
	Unadjusted OR (95%CI)	p	Adjusted OR (95%CI)*	p	Unadjusted OR (95%CI)	p	Adjusted OR (95%CI)^†^	p
Gender								
Female								
Male	1.08 (0.44–2.69)	0.87	1.97 (0.75–5.19)	0.17	1.08 (0.44–2.69)	0.87	1.06 (0.41–2.73)	0.91
Number of employment sites								
1								
>1	0.63 (0.40–1.00)	0.052	0.59 (0.34–0.99)	0.045	1.28 (0.80–2.04)	0.30	1.34 (0.81–2.21)	0.26
Main place of employment								
Other than hospital								
Hospital	1.28 (0.86–1.90)	0.22	1.33 (0.85–2.08)	0.21	1.07 (0.71–1.59)	0.76	1.17(0.75–1.81)	0.50
Age								
≤ 35								
> 35 to 42	2.40 (1.55–3.72)	<0.001	2.55 (1.60–4.07)	<0.001	0.95 (0.61–1.47)	0.81	1.03 (0.65–1.63)	0.90
> 42 to 47	1.75 (1.10–2.78)	0.018	2.09 (1.27–3.44)	0.004	0.78 (0.48–1.25)	0.30	0.83 (0.51–1.37)	0.47
> 47	1.75 (1.12–2.74)	0.014	2.65 (1.60–4.40)	<0.001	0.99 (0.63–1.55)	0.95	1.25 (0.76–2.05)	0.38
Location of medical center								
rural								
urban	0.71 (0.52–0.98)	0.036	0.81 (0.57–1.15)	0.24	0.71 (0.51–0.98)	0.038	0.68 (0.48–0.96)	0.030
Duration of Internet use								
no use or ≤ 1 year								
>1 to 2 years	1.31 (0.65–2.66)	0.46	1.07 (0.51–2.25)	0.87	1.38 (0.65–2.92)	0.40	1.34 (0.62–2.91)	0.46
>2 to 5 years	2.49 (1.53–4.05)	<0.001	2.73 (1.62–4.60)	<0.001	1.92 (1.15–3.22)	0.013	1.81 (1.06–3.11)	0.031
>5 to 10 years	2.14 (1.32–3.46)	0.002	2.91 (1.71–4.94)	<0.001	1.89 (1.13–3.15)	0.015	1.73 (1.01–2.99)	0.048
> 10 years	2.10 (1.19–3.70)	0.011	2.94 (1.58–5.46)	0.001	2.42 (1.34–4.37)	0.003	2.29 (1.23–4.26)	0.009

^#^ Binomial variable originating from initial variable used in the questionnaire after collapsing five categories of the Likert scale (“1” = “agree” corresponding with”strongly agree” or”rather agree” and”0” = “other opinion” corresponding with”strongly disagree”,”rather disagree” or”neither agree nor disagree”)

* Hosmer-Lemeshow test, chi^2^ = 8.18, df = 8, p = 0.42

^†^ Hosmer-Lemeshow test, chi^2^ = 5.52, df = 8 p = 0.70

Abbreviations: OR–odds ratio, 95%CI–95% confidence interval

In multivariate logistic regression model, the duration of the use of the Internet and age maintained their effect on the opinion about the reliability of health-related information on the Internet (please see Table 4). Interestingly, the effect of the employment in a medical center classified as rural or urban was no longer statistically significant. However, the impact of the number of sites of employment reached statistical significance–more than one site of employment was related to lower belief in the reliability of health-related information on the Internet (OR = 0.59, 95%CI: 0.35–0.99).

The univariate model revealed that the acceptance of Internet/e-mail use for the communication between a patient and a physician was lower among respondents employed in medical centers classified as urban (OR = 0.71, 95%CI: 0.51–0.98) and higher among respondents who had used the Internet for longer periods. OR and 95%CI for comparisons between the group of respondents who did not use the Internet or had used it for a period not longer than 1 year and three groups of the longest duration of Internet use were 1.92, 1.15–3.22, 1.89, 1.13–3.15 and 2.42, 1.34–4.37 respectively. The location of the medical center and duration of Internet use maintained their impact on the acceptance of the use of the Internet/e-mail for communication between a patient and a physician in the multivariate model. Detailed results of univariate and multivariate modeling are shown in Table 4.

The predictors of the nurses’ acceptance of patient access to their Internet-based medical record, in both the univariate and multivariate models included the location of the medical center, type of the main site of employment and duration of Internet use (Table 5). In the univariate model, the acceptance was lower among respondents employed in hospital centers located in urban areas (OR = 0.59, 95%CI: 0.43–0.81), and among those who declared a hospital as being their main place of employment (OR = 0.64, 95%CI: 0.43-.96). It was higher among those who had used the Internet for longer periods (OR at least 2.45 for comparisons between the group of the shortest Internet use and groups of longer duration of the Internet usage). These effects were also seen in the multivariate logistic regression model.

**Table 5:** The results of univariate and multivariate logistic regression modeling for acceptance of patient access to their Internet-based medical record and making recommending use of telemonitoring to family members with chronic disease.

Variable	Patient should have Internet-based access to his/her medical record^#^ (‘agree’ vs. ‘other opinion’)				Would you recommend use of an Internet-based system for monitoring disease to a person from your close family?^#^ (‘agree’ vs. ‘other opinion’)			
	Unadjusted OR (95%CI)	p	Adjusted OR (95%CI)*	p	Unadjusted OR (95%CI)	p	Adjusted OR (95%CI)^†^	p
Gender								
Female								
Male	1.53 (0.58–4.03)	0.39	2.00 (0.72–5.53)	0.18	1.61 (0.63–4.14)	0.32	1.71 (0.64–4.62)	0.29
Number of employment sites								
1								
>1	1.10 (0.68–1.77)	0.70	1.03 (0.61–1.73)	0.93	1.39 (0.87–2.22)	0.17	1.24 (0.75–2.06)	0.41
Main place of employment								
Other than hospital								
Hospital	0.64 (0.43–0.96)	0.031	0.61 (0.39–0.96)	0.034	0.83 (0.56–1.21)	0.33	0.79 (0.52–1.21)	0.28
Age								
≤ 35								
> 35 to 42	1.32 (0.85–2.05)	0.21	1.33 (0.83–2.12)	0.24	1.38 (0.90–2.12)	0.14	1.61 (1.02–2.54)	0.040
> 42 to 47	1.03 (0.65–1.63)	0.91	1.00 (0.61–1.63)	0.99	1.06 (0.67–1.66)	.82	1.22 (0.75–1.98)	0.42
> 47	1.20 (0.77–1.88)	0.42	1.42 (0.86–2.33)	0.17	1.08 (0.70–1.67)	0.73	1.35 (0.83–2.18)	0.23
Location of medical center								
rural								
urban	0.59 (0.43–0.81)	0.001	0.57 (0.40–0.80)	0.001	0.87 (0.64–1.18)	0.37	0.89 (0.64–1.26)	0.52
Duration of Internet use								
no use or ≤ 1 year								
>1 to 2 years	3.13 (1.50–6.53)	0.002	2.46 (1.14–5.27)	0.021	2.03 (1.01–4.09)	0.048	1.61 (0.78–3.32)	0.20
>2 to 5 years	2.78 (1.71–4.51)	<0.001	2.49 (1.49–4.17)	0.001	1.95 (1.21–3.17)	0.006	1.85 (1.12–3.07)	0.017
>5 to 10 years	2.51 (1.56–4.04)	<0.001	2.54 (1.51–4.26)	<0.001	2.23 (1.39–3.60)	0.001	2.20 (1.32–3.66)	0.003
> 10 years	2.45 (1.39–4.32)	0.002	2.22 (1.21–4.05)	0.010	2.89 (1.63–5.12)	<0.001	2.93 (1.60–5.36)	0.001

^#^ Binomial variable originating from initial variable used in the questionnaire after collapsing five categories of the Likert scale (“1” = “agree” corresponding with”strongly agree” or”rather agree” and”0” = “other opinion” corresponding with”strongly disagree”,”rather disagree” or”neither agree nor disagree”)

* Hosmer-Lemeshow test, chi^2^ = 10.87, df = 8 p = 0.21

^†^ Hosmer-Lemeshow test, chi^2^ = 4.49, df = 8 p = 0.81

Abbreviations: OR–odds ratio, 95%CI–95% confidence interval

The univariate logistic regression models indicated that the duration of the Internet use was the only predictor for making recommendation for the use of a telemonitoring system by a family member suffering from chronic condition (OR for all four comparisons from 1.01 to 1.63). This effect was also maintained in the multivariate model, however, a significant difference was also found for the age of respondents. The respondents from the older age group of age (>35 to 42 years) were more inclined to make a recommendation about telemonitoring of relatives than those from the youngest group (OR = 1.61, 95%CI: 1.02–2.54). The difference was not seen in the case of the two oldest age groups. The results of both logistic regression models are presented in Table 5.

## Discussion

In this study, the opinions of nurses about three types of ehealth services potentially important for patients with chronic diseases were assessed. These included the use of Internet or e-mail for communication between a patient and a physician, patient telemonitoring and patients having access to their Internet-based medical record. Furthermore, their opinions about the reliability of health-related Internet-based information were explored. The mean age of respondents was 40 years which corresponds closely to the national ratio of group of health care professionals in Poland. According to a report issued by the National Chamber of Nurses and Midwives in 2009, the age of 35.8% of all nurses in Poland was in the range of 36–45 years. In 2009, the mean age of a nurse in Poland was 44.2 years [48].

Computer use was confirmed by approximately 92% and Internet use by nearly 83% of the respondents. Respondents’ Internet usage was decidedly higher than that of the general population in Poland (62% of the population 17-74-year-olds, according to EUROSTAT) [49]. However, the nurses’ use of the Internet was considerably lower than among physicians (the physicians’ usage rate reached 100%) [31]. Furthermore, 44% of respondents had not used the Internet longer than five years.

According to a Report from a large study carried out by Hegney et al. among nurses in Australia, having no computer or Internet experience was admitted by only 1.9% and 7.1% of nurses respectively [50]. The survey carried out by Gilmour et al. in 2011 among nurses employed in medical wards in New Zealand demonstrated that 92.8% of respondents had access to online health information at work and at home [51]. In a survey from 2006, Lupiáñez-Villanueva et al. found that from a large group of nurses, who were members of the Nurses Association of Barcelona, only 4.5% indicated that they never or hardly ever used the Internet outside the workplace [52]. The survey presented in our paper was performed in 2011 and 2012, and, the percentage of nurses who did not use the computer and Internet at all are still much higher than in the studies performed by Hegney et al. and Lupiañez-Villanueva several years earlier. This comparison highlights the fact that Poland lags behind other countries with development of an ehealth environment.

Among the three types of ehealth applications asked about, the respondents revealed the lowest acceptance for electronic communication between a patient and a physician (only about 38% positive responses). The high level of reservation concerning the use of electronic communication for contact between patients and physicians is apparently shared by other health care professionals. This feature was also the ehealth application with the lowest level of acceptance among physicians in Poland [31].

A total of 67.8% of respondents supported patients having access to their medical record via the Internet and 51.9% would recommend the use of telemonitoring to family members suffering from chronic diseases.

In the study by Hegney et al., the percentage of responses agreeing with benefits coming from national electronic health records reached 67.5% [50]. Many other authors reported nurses expressing **c**onsiderable reservations toward electronic health record systems, especially after these systems were implemented [53–55]. These opinions were summarized by Sassen [56]. However, nurses’ attitudes toward patient access to electronic health record among nurses were not studied extensively. In our study, the level of acceptance for patient access to medical records is relatively high.

The use of telemonitoring services in the Polish health care system remains very low. There is no recent data available regarding this aspect of ehealth development in Poland. The results of the European study *eHealth Indicators* reported that in 2007 no telemonitoring services were offered by general practitioners to their patients [30]. It seems that currently tele-electrocardiography (tele-ecg) is the main type of telemonitoring application available to patients on routine basis in Poland [57]. Other telemonitoring applications are rare. Even though telemonitoring is not commonly available for chronic patients, the acceptance of this type of ehealth service was relatively high among nurses.

The acceptance for all three types of ehealth applications was consistently influenced by the variable combining the information on the Internet use and its duration. Acceptance was higher among respondents who had used the Internet for longer periods as compared to those who did not use the Internet or used it for a period not surpassing 1 year. This effect was maintained consistently both, in the univariate and multivariate logistic regression models. Other factors, which exerted an effect on acceptance included location of employment (two of three types of applications), and the type of main employment site (one of three applications). Interestingly, respondents employed in hospital centers classified as urban were less prone to accept the use of Internet for communication between a patient and a physician and for access to medical record by patients. Furthermore, respondents who declared their place of main employment as a hospital were also less inclined to accept patients being able to access their medical records.

More than half the respondents (54.6%) were convinced that health-related information available on the Internet is usually reliable. The study by Gilmour et al. carried out among postgraduate nursing students in New Zealand revealed that 49.1% of their respondents believed only some Internet-based health information and 46.6% most information [58]. The study carried out by the same team in 2011 revealed that as many as 49.8% of respondents believed only some health-information on the Internet [51]. In our study, the belief in reliability of such information was higher among respondents using the Internet for longer periods, employed in medical centers located in rural areas and belonging to older age groups. The finding that older respondents showed higher trust in the Internet-based health-related information was unexpected, but evidently younger respondents who are more frequently Internet users, are also more critical about its content.

As far as limitations of the study, one consideration is that the sample of respondents was not fully representative and that selection of hospital centers for the survey was not randomized, but based on the first positive responses by representatives of management staffs approached. Furthermore, a considerable number of returned questionnaires were excluded from the study after a quality check, mainly due to a high frequency of missing values. Finally, the questionnaire included only a limited number of variables addressing socioeconomic characteristics of respondents and in consequence, potentially important factors influencing attitudes toward ehealth solutions could have been omitted such as, e.g. income status.

## Conclusions

Younger nurses, employed in health care centers located in urban areas, demonstrated higher computer and Internet use. Simultaneously, younger age and having more than one place of employment were related to lower trust in health-related resources available on the Internet. The acceptance of patient use of ehealth applications was consistently dependent upon duration of Internet use by respondents. Furthermore, acceptance of e-mail communication between patient and physician, and patients having Internet-based access to their medical record was higher among nurses employed in health care centers located in rural areas. Finally, this study shows that aside from the pressing need to build an ehealth infrastructure and make it an integral part of health care system, there is a need of educating and training of nursing personnel to enable their involvement in the ehealth implementation efforts.

## Supporting Information

S1 File Data set used for the analysis. (XLSX)
